# Value of Face-to-Face Interactions Between Clinician-Educators and Patients or Students to Improve Health Care Education

**DOI:** 10.2196/humanfactors.9859

**Published:** 2018-04-20

**Authors:** Manisha Singh

**Affiliations:** ^1^ Division of Nephrology Department of Internal Medicine University of Arkansas For Medical Sciences Little Rock, AR United States

**Keywords:** medical education, human factor, value in human interactions

## Abstract

The power and outreach of the media is enormous and has restructured our society today; the author acknowledges the impact and appreciates the outreach. However, I question the relative lack of focus on physical human interactions and express concern over future training efforts. I have compared and attempted to highlight the components of two interaction scenarios: those of teacher-student, and those of physician-patient. The physician-educators need to generate a discussion regarding the value of each interaction. As a teacher, there is value in online classrooms, and a different value in face-to-face interactions. Similarly, a physician can have major outreach impact by online tele-medicine and tele-education efforts, but in some instances, may need to have the human, physical interaction with the patient. The value of these interactions depends on the roles in which these interactions are experienced. Medical education training must incorporate an understanding of the unique value of different interactions.

## Observations

The “human factor” is the essence of teaching and learning as well as that of clinical care. With the advent of media, we need to examine its role as it is taught and learnt, expressed as “face-to-face” versus “using technology” as a medium of interaction. The value of the medium used for an interaction has to be assessed based on the outcome expected from that interaction. In some circumstances, the use of media is very helpful. Conversely, sometimes media takes away value from the purpose of the interaction.

## Questions

### Experience of classroom teaching

I arrive early, formally dressed, ready with a PowerPoint presentation. The PowerPoint consists of information most relevant for exams, with example questions and case-based presentations. I walk into a class of 10 students and teach from the podium. There is a live feed on. The other 155 students are at different places, logging in from their places of choice. Many review the lecture videos later. After class, they critique my lecture for relevance to the board preparation. My future presentations will be based on the critiques from these students. The same topics have been covered well by leading experts in the area with freely available videos. Between the textbooks and internet resources, where is the need of an educator standing in class on a particularly rainy morning?

Is the experience of the students in class different from those online?

My experience in the clinic is also similarly thought provoking with the advent of media.

### Patients in a subspecialty clinic

The patient can be treated without meeting the doctor in some cases. For instance, for a patient with chronic kidney disease we can order laboratory tests and the primary care physicians’ note can tell us about the exam findings needed to prescribe appropriate medications. For routine subspecialty assessments we may not need a physical exam. The vitals can be texted in. An effective, guideline-based management plan can be put in place without any person-to-person interactions. The patient can be emailed the medication side effect list and get an online consent, which may be a safe process as it related to liability.

The subspecialty can do away with most barriers that lead to conflicts. The biases of nationality, gender, and language can all be transcribed over. The human factor can thus be eliminated entirely in both of these scenarios. However, there is some value in each interaction: using media or not using media; with or without the human factor.

## The Human Factor

We, as people, have an identity of self and also an identity in the roles we play. Each role comes with its society-approved behavior patterns: roles of spouses, children, or being parents; roles of identifying with a particular gender, race, or nationality. Each role comes with its own set of expected behaviors, attire, and stage setting. The role of a physician comes with props like the white coat and the stethoscope, the stage setting of a clinic or hospital, and in recent advances, tele-health support. The role of a student may come with a recognition of some degree of need for training and accepting a person as a teacher (in the setting of a classroom or through online presence).

My pedagogy mentors emphasized a balance of head, hands, and heart when I started my career in clinical education. While the head and hands are approachable through media, we may need to look closer at the heart for training and treatment purposes. Regardless of the modality of interaction (face-to-face or via media), the perceived roles of human interactions can vary between those of student-teacher, physician-patient, and vendor-consumer.

Each role has a set of rights and duties assigned, and also some combination of the following individual components: required knowledge (head), the required skills (hands), and the required attitudes that go with this (heart). An effective combination of these factors is what constitutes the human factor: the person you meet.

### Student-Teacher Scenario

The teacher is more than a container/dispenser of information. The personality of an educator is unique. The scales we use to assess the caliber of a teacher uses only objective data: how well the students do on exams and their assessment of the course content with respect to national exams. The subjective experience of being a student in classroom, facing a teacher, and the outcome of this interaction is a human relationship; its value is very hard to express/assess. The student learns more than the subject. The teacher, in turn, gets value in that experience. When a teacher teaches in class, he/she brings with that PowerPoint presentation, a human factor which responds and relates to the student.

Regarding the content of education material, recent advances with tele-education have expanded educational outreach beyond imagination [1]. Initially we could have critiqued that the content available is not peer reviewed and lacks the authenticity of experts opining on the content [2]. This line of thinking has changed completely with online discussions with experts on various platforms, as seen with online journal clubs, media groups, and scientific communities. There is an admirable turnaround time of a scientist’s question being answered in the digital world.

### Physician-Patient Scenario

The physician that is present in the clinic has a relationship with a patient he/she faces in a patient-doctor relationship, which is different from communicating with the same patient through media. Although tele-education and tele-medicine are enormously helpful in many cases, there is something to be said for the personal interaction.

### What is the Value of Holding a Hand, Making Eye Contact, and Talking to Another Person?

The physician, much like the teacher, aspires to “make the patient feel better.’’ There is a sense of aspiring for a higher purpose in life. Most physicians in the role of “the doctor” care deeply about the best state of health possible for the patient in accordance with the patient's own challenges. To achieve this, they train to the extent of their abilities and challenge limitations. Their interactions are based on the roles perceived by each. A physician can see himself in the role of a caregiver, or that of a vendor, regardless which form of communication he/she chooses to use. A patient can see him/herself as a person needing comfort or a consumer, again, regardless of how he/she is communicating with the physician. These perceived roles can be seen similarly in any situation, be it personal interaction or through a digital presence. The human factor introduces a relationship between roles. We need to understand the roles of the patient-doctor versus roles of the vendor-consumer. In any scenario, either relationship may be perceived.

If the physician is seen in the role of a vendor, the patient assumes the role of a consumer, and must look for a certain specific objective value in the encounter [3]. A vendor's motivation is different than that of a physician. In this scenario, there is not much value in the time spent sitting silently holding a patient's hand after delivering bad news. A 15-minute encounter, encompassing a full review of systems, an exam, review of lab results, and assessment and management plan, has no financial incentive to add to the question, “How do you feel today?” The best gain scenario is to identify a problem list quickly and focus on the exam and a treatment plan, minus the human-factor.

The patient, in the role of a patient, is vulnerable and expects to be comforted. Armed with internet knowledge, the patient may require more detailed discussions than previously anticipated [4]. In the role of the consumer, the patient is able to critique the physician for “professionalism” which is a very subjective interpretation. For the “consumer” the option of using media may present the best-case scenario, while the role of a “patient” may need a human interaction with a person in the physical role of a doctor.

### Unifying Concepts

The role of basic courtesy is crucial in either case, but is there any added value to physically meet a teacher/physician? Is an email the same thing as meeting someone in person? How does Skype compare to face-to-face meetings? Are we gaining in economics and losing in humanities?

## Will Either Situation Add Value to The Objective of That Interaction?

Where there is no health care, or in areas where it is difficult to obtain subspecialty quality care, tele-medicine certainly is the most valuable tool. Similarly, for lectures, online tools are very valuable for distant outreach. However, there is still a place for training in human interactions for many scenarios in which the value is in the absolute human interaction.

There are places in which this is helpful, and there may be places that this is time consuming, redundant, and expensive. In changing times, with the power and outreach of media, a discussion that fully incorporates the advantages of media and the possibilities of including the human factor for an effective classroom and clinical experience is warranted.
